# Immunomodulatory Role of Surfactant Protein-D in a Transgenic Adenocarcinoma of Mouse Prostate (TRAMP) Model

**DOI:** 10.3389/fimmu.2022.930449

**Published:** 2022-07-07

**Authors:** Kasturi Ganguly, Uday Kishore, Siddhanath M. Metkari, Taruna Madan

**Affiliations:** ^1^ Department of Innate Immunity, Indian Council of Medical Research (ICMR)- National Institute for Research in Reproductive and Child Health, Mumbai, India; ^2^ Biosciences, College of Health and Life Sciences, Brunel University London, Uxbridge, United Kingdom; ^3^ Department of Veterinary Medicine, United Arab Emirates (U.A.E) University, Al Ain, United Arab Emirates; ^4^ Indian Council of Medical Research (ICMR)- National Institute for Research in Reproductive and Child Health, Mumbai, India

**Keywords:** prostate cancer, SP-D, TRAMP model, immunomodulaion, innate immunity, immunogenic cell death (ICD), collectin

## Abstract

Surfactant protein D (SP-D), a pattern recognition molecule, is emerging as a potent anti-tumoural innate immune defense molecule in a range of cancers. Previously, SP-D expression was found to be significantly downregulated at the malignant sites of human prostate adenocarcinoma and associated with an increasing Gleason score and severity. We recently reported selective induction of intrinsic apoptosis by a recombinant fragment of human SP-D (rfhSP-D) in the human Prostate cancer (PCa) biopsy explants and cells with glucose regulated protein of 78 (GRP78) as one of the key interacting partners. The present study evaluated the expression of SP-D in early and advanced stages of PCa using transgenic adenocarcinoma of mouse prostate (TRAMP) model. Both early and late stages of PCa showed significantly decreased SP-D mRNA expression and increased proteolytic degradation of SP-D protein. Systemic and tumoural immunophenotyping of TRAMP model revealed increased serine proteases producing granulocytes and polymorphonuclear myeloid-derived suppressor cells (PMN MDSCs) in the late stage; the serine proteases secreted by these cells could be involved in the degradation of SP-D. Susceptibility of rfhSP-D to elastase-mediated proteolysis provided the rationale to use an elastase-inhibitor to sustain intact rfhSP-D in the tumour microenvironment. The study revealed an immunomodulatory potential of rfhSP-D and elastase inhibitor, sivelestat, to induce macrophage polarization towards M1 with downregulation of PMN MDSCs in *ex-vivo* cultured TRAMP tumours. Furthermore, rfhSP-D induced immunogenic cell death in murine PCa cells and in TRAMP explants. The findings highlight that SP-D plays an anti-tumourigenic role in PCa by inducing immunogenic cell death and immunomodulation while the prostate tumour milieu adversely impacts SP-D by inhibiting its transcription, and enhancing its proteolytic degradation. Transformation of an immunologically “cold tumour” into a “hot tumour” implicates therapeutic potential of rfhSP-D in PCa.

## Introduction

Prostate cancer (PCa) is one of the deadliest cancer in men with second highest incidence rate globally (1). Although its incidence in Asia is much lower as compared to the U.S.A. and the Europe, the cancer statistics of China demonstrates a stably increasing trend of the incidence and mortality due to PCa (2). The conventional anti-cancer treatments, mainly androgen-deprivation therapy (ADT), are efficacious by providing a high rate of progression-free survival. However, 30–50% of patients experience recurrence and eventually progress to metastatic castration-resistant prostate cancer (mCRPC) (3). Tumour antigen-specific adaptive immunotherapies have improved clinical outcomes and revolutionized the treatment paradigm in many hematological and solid malignancies (4–6). These approaches, however, have been largely unsuccessful for mCRPC, owing to the “cold tumour” microenvironment which is characterized by immunosuppression, low tumour mutation burden (TMB), low immunogenicity, minimal T-cell infiltration, and downregulated MHC-I molecules (3–5, 7). Lack of pre-requisites for successful prostate cancer-specific adaptive immune therapy has given rise to a sub-category of poor responders (6). This necessitates consideration of tumour antigen-independent, innate immune responses mediated by pattern recognition receptors (PRRs) as an alternative or complementary means to target cancer cells.

Surfactant protein D (SP-D), a soluble pattern recognition receptor (PRR) belonging to carbohydrate-binding lectins or C-type lectin receptors (CLRs) family, plays a key role in host defence. They serve as an innate immune molecule in the control of inflammation, infection, allergy, and cancer (8, 9). Structurally it is composed of an N-terminal cysteine-rich domain, a triple-helical collagen domain consisting of Gly-X-Y repeating triplets, an α-helical coiled coil neck region, and C-terminal carbohydrate recognition domain (CRD) or C-type lectin domain (10). Depending on its structural oligomerization and interaction with cell surface receptors, SP-D performs a dual immune surveillance role by triggering either pro-inflammatory or anti-inflammatory signaling (11, 12). Though it was initially considered as a lung collectin due to its association with pulmonary surfactant, the immunological roles of SP-D at extrapulmonary and mucosal sites are now well established (10, 13). In rat prostate, upregulation of SP-D in response to *Escherichia coli* infection as well as the presence of SP-D in human prostate and prostatic secretions suggested that SP-D could be involved in prostate immunity (14). Kankavi et al. first reported decreased expression of SP-D and SP-A proteins at the malignant site of human prostate adenocarcinoma compared to non-malignant areas in the same tissue sections (15). The potential of SP-D acting as an immunosurveillance molecule in PCa was subsequently demonstrated by our group with the use of a recombinant fragment of human SP-D (rfhSP-D) comprising 8 Gly-X-Y repeats trimeric neck and CRD that selectively induced intrinsic apoptosis in human prostate biopsy explants as well as in human prostate cancer cells including androgen-responsive LNCaP and androgen-resistant PC3 (16). In-silico and *in-vitro* analyses of the membrane interactome of rfhSP-D in PC3 cell line revealed that rfhSP-D bound glucose regulated protein of 78 kDa (GRP78) through its CRD region and interfered with the pro-survival signaling (17). rfhSP-D has also been shown to differentially induce TNF-α and Fas-mediated pro-apoptotic signaling pathway and activate caspase cascade, and thus, induce apoptosis in pancreatic ductal adenocarcinoma (PDAC) cell lines such as Panc-1 Capan-2, and MiaPaCa-2 as well as in an ovarian cancer cell line SKOV3 (18, 19). rfhSP-D also inhibited epithelial-to-mesenchymal transition (EMT) by inhibiting transforming growth factor-β (TGF-β) pathway and downregulating mesenchymal specific markers, such as zinc finger E-box binding homeobox 1 (Zeb1), Snail, and Vimentin in PDAC cell lines such as Panc-1, and MiaPaCa-2 (20). SP-D has also been shown to interrupt epidermal growth factor (EGF) signaling by blocking the EGF–EGFR interaction in human lung adenocarcinoma A549 cell line (21). Proteomics analysis of rfhSP-D-treated eosinophilic cell line, AML14.3D10 line resulted in oxidative stress-mediated apoptotic signaling with downregulation of HMGA1, a pro-survival factor (22).

With established potent anti-cancer activity of rfhSP-D at the cellular level mediated through several molecular mechanisms, it is important to explore therapeutic implication of rfhSP-D in the murine models of PCa. In context to this, it has been observed that several serine proteases, mainly neutrophil elastase (NE), are known to cleave SP-D at the CRD and give rise to stable non-functional degraded fragment of ~35kDa (23, 24). Thus, it is of great interest to analyze the susceptibility of SP-D to proteolysis in NE-rich PCa microenvironment.

In this study, using a transgenic adenocarcinoma mouse prostate (TRAMP) model that is a spontaneous PCa model closely resembling the development and stages of human PCa, we evaluated the expression of SP-D in early and advanced stage of tumour progression. A stage-dependent downregulated expression of SP-D in tumour was observed possibly due to increased proteolysis of the protein. In addition, susceptibility of rfhSP-D to elastase, a serine protease, highlighted the importance of a new therapeutic strategy that maintained the stability of rfhSP-D in elastase-rich PCa tumoural microenvironment (TME). Furthermore, immunosuppressive TME can be altered with the exogenous treatment of rfhSP-D combined with elastase inhibitor in the *ex vivo* cultured TRAMP tumours. For the first time, we show that, rfhSP-D can induce immunogenic cell death (ICD) in murine PCa cells and in TRAMP explants. Together, our results provide a rationale for exploiting the therapeutic potential of rfhSP-D to treat immunologically unresponsive tumours.

## Materials and Methods

### Transgenic Adenocarcinoma of Mouse Prostate (TRAMP) Model

All animal experiments were approved by the Institutional Animal Ethics Committee (IAEC No. 09/18). Mice representing Transgenic Adenocarcinoma of Mouse Prostate (TRAMP) were purchased from the Jackson Laboratory (C57BL/6-Tg (TRAMP)8247Ng/J); the model closely resembles the pattern of disease progression and stages of human PCa. Mice hemizygous to the transgene, where prostate-specific rat probasin promoter is used to drive expression of the Simian Virus 40 large tumour T antigen, spontaneously develop progressive forms of PCa from pre-cancerous prostatic intraepithelial neoplasia (PIN), well-differentiated carcinoma (WD), poorly differentiated adenocarcinoma (PD) of the prostate gland, and finally metastasis (25). Hemizygous TRAMP mice were maintained in C57BL/6 background as the stock colony, whereas TRAMP mice were bred to non-transgenic normal FVB/NJ mice to obtain non-transgenic and hemizygous transgenic [C57BL/6 TRAMP x FVB/NJ] F1 male progenies that were used for the experiments. Prior to the start of the experiments, mice were allowed to acclimatize for one week. During the course of the experiment, mice were monitored weekly for general health, food consumption, and body weight. Mice were kept under controlled temperature, and humidity condition with 12-hour light: 12-hour dark cycle and were fed *ad libitum*.

### Genotyping of TRAMP Mice

TRAMP mice were genotyped for confirmation as reported (26). Briefly, tail DNA was isolated using RED Extract-N-Amp Tissue PCR Kit (Sigma, USA). The primers recommended by the Jackson Laboratory protocol for Tg (TRAMP) 8247Ng stock mice, specific for rat probasin gene (Transgene; Tg), and as internal positive control (IPC) primers specific for beta-actin gene, were used for PCR. The PCR end products were run on a 2% agarose (GeNei, India) gel to confirm the presence or absence of the transgene.

### RNA Isolation and Quantitative Reverse Transcription PCR (RT-qPCR) Analysis

Total RNA was isolated from prostate tumour or control dorsal lobe prostate using the TRIzol reagent (Ambion by Life technologies, USA) and quantified in multimode plate reader (BioTek Synergy H1, USA). 1 μg RNA was reverse transcribed using Superscript III first-strand synthesis kit (Invitrogen, USA). RT-qPCR was performed using Bio-Rad CFX 96™ Thermal Cycler machine (Bio-Rad, USA) with 1.25 μl of the first-strand cDNA for each 25 μl real time PCR reaction using SYBR green (BioRad, USA) and forward and reverse primers. The semi-quantitation of the relative target mRNA was carried out using the ∆∆CT comparative method after normalizing with GAPDH abundance in the same sample. The specificity of each amplified product was validated by analysing their melt curve and by visualizing the band size of the amplified products on a 2% (w/v) agarose gel. The assay was performed in triplicates and the mean value was taken for further calculations. The primers were designed from NCBI Primer BLAST Software. Primer pair sequences, amplicon sizes, and their annealing temperatures are listed in **Table 1**.

**Table 1:** Details of murine primers.

Target	Forward primer (5’ → 3’)	Reverse primer (3’ → 5’)	Amplicon size (bp)
Transgene	TACAACTGCCAACTGGGATG	CAGGCACTCCTTTCAAGACC	264
Internal Positive Control (β-actin)	CTGTCCCTGTATGCCTCTGG	AGATGGAGAAAGGACTAGGCTACA	415
SP-D	GCAGGACATGCTGCCCTTTCT	ACCCTTCTCACCCCGTGGACC	196
SP-A	ACCTGGATGAGGAGCTTCAGACTC	TGCTTGCGATGGCCTCGTTCT	225
MBL-A1	ACCAGGTCAAGGGCTCAGGGG	TGCCAGCTTCTCCTCAATGGCTC	141
GAPDH	CTGAGCAAGAGAGGCCCTATCC	CTCCCTAGGCCCCTCCTGTT	101

### Immunohistochemistry (IHC) Analysis

Formalin-fixed prostate tumours of TRAMP mice or dorsal lobe of control prostate tissues were paraffin-embedded, sectioned, and mounted on poly-L-lysine coated slides. 5 μm sections were deparaffinized in xylene and gradually rehydrated in a methanol gradient of 100%, 90%, 70%, 50%, 30%, and distilled water, each for 5 min. Blocking of endogenous peroxidase activity was done by immersing the rehydrated sections in a 3% hydrogen peroxide solution for 30 minutes. To improve antigen retrieval, the slides were boiled in sodium citrate buffer (pH 6.0) in a microwave. After the slides cooled down, they were washed with PBS, and any non-specific binding was blocked with 5% BSA in Tris buffer saline with Tween 20 (TBS-T) for an hour at room temperature (RT). The slides were incubated with a rabbit polyclonal primary antibody against mouse SP-D (Ab203309, Abcam, UK, 1:50 dilution in PBS) overnight at 4^◦^C in a humidified chamber. For negative control, tissue sections were treated with the antibody diluent, PBS alone, without primary antibody. Next day, slides were washed in PBS, incubated with horseradish peroxidase (HRP)-conjugated polyclonal goat anti-rabbit IgG (ab205718, Abcam, 1:15000 dilution in PBS) at RT for an hour. Visualization was done using diaminobenzidine (DAB) (Sigma, USA) incubation for 10 min. Next, the nuclei were counterstained with Mayer’s haematoxylin (SRL, India), followed by dehydration and mounting using DPX (SRL, India). Images were taken with Leica AS LMD microsystem at 40× magnification. The immunostaining was quantified using the open-source plugin, IHC profiler compatible with the open resource digital image analysis software, ImageJ, followed by calculation of the H score as previously described (27). For each tissue section, a minimum of three to four areas were scanned and the average H score was calculated and plotted as the mean ± SEM.

### Total Protein Isolation and Immunoblot Analysis

TRAMP prostate tumour tissue or dorsal lobe of the prostate from control mice (~100 mg) were minced and homogenized in RIPA lysis buffer (Millipore Sigma, USA) with protease inhibitor cocktail (Thermo Fisher Scientific, USA). The homogenate was incubated for 30 min on ice followed by centrifugation at 14,000× g for 30 min at 4^◦^C. The supernatant was collected and the total protein content was determined by BCA Protein Assay (Thermo Fisher Scientific, USA). 30 μg of protein was loaded in each lane and resolved on 12% v/v SDS-PAGE under reducing conditions. The resolved proteins were transferred onto an activated polyvinylidene fluoride (PVDF) membrane (Pall Corporation, USA), then blocked with 5% BSA in TBS containing 0.1% (v/v) Tween-20 (GeNei, India) (TBS-T) for 1h at RT. The membrane was probed overnight at 4°C with a rabbit polyclonal primary antibody raised against mouse SP-D (ab203309, Abcam, USA, 1:500 dilution in PBS) and a rabbit monoclonal antibody against mouse GAPDH (2118S, Cell signalling Technology, 1:1000 dilution in PBS) as the reference control. Next day, the blots were washed in TBS-T and probed with HRP-conjugated polyclonal goat anti-rabbit IgG (ab205718; Abcam, 1:15000 dilution in PBS) for 1 h at RT. Finally, the protein bands were detected with ECL Western Blotting Substrate (Thermo Fisher Scientific, USA). Quantitative densitometric analysis of the protein bands corresponding to intact SP-D and cleaved SP-D was conducted using ImageJ software and the band intensity was normalized using corresponding GAPDH as the loading control. The quantitative data were presented as mean ± SEM.

### Immunophenotyping by Flow Cytometry

For phenotypic characterization of circulatory and prostate-resident immune cells, mice were euthanized, prostate tissue and whole blood (collected by trans-cardiac bleeding into K_2_EDTA tubes to prevent clot formation) were collected, and stained with nine-colour combinations of fluorescently labelled monoclonal antibodies. Single cell suspensions were prepared as reported previously (28). Briefly, prostate tumour tissues from TRAMP or control prostate were harvested, washed with ice-cold PBS, enzymatically digested with collagenase IV (Gibco, USA) at 37°C for 2 h, and centrifuged at 400 x g for 5 min. The samples were treated with warm 0.05% trypsin-EDTA and 500U DNase I (Sigma, USA). The enzyme digested suspensions were passed through a 70-μm cell strainer (Corning, USA), centrifuged for 5 min, and re-suspended in FACS staining buffer [phosphate-buffered saline (PBS) with 0.2% foetal bovine serum (FBS)]. Dissociated cells were then collected, washed, counted, and distributed in tubes (10^6^ cells/ml) for multicolour flow cytometry. The fresh EDTA-collected blood cells and single-cell suspensions (200 μl) were incubated with fluorescently labelled monoclonal antibodies for 20 min at RT. In the blood samples, RBCs were lysed using BD Pharm lyse (BD Biosciences) and washed twice with staining buffer (PBS with 0.2% FBS). Antibodies utilized for flow cytometry are listed in **Table 2**; antibodies were individually titrated to determine optimal staining dilutions. Data acquisition was performed on BD ACCURI C6 flow cytometer (BD Biosciences) where at least 50,000 events gated on the CD45^+^leukocyte population were acquired. Anti-rat Ig, κ/Negative Control compensation beads (BD Biosciences) were used to set compensation parameters. Fluorescence minus one (FMO) and unstained controls were used to identify and gate cells. Data analysis was carried out using FlowJo v10.8.1(Tree Star Inc., Oregon, USA). A representative flow cytometry gating strategy is displayed in **Figure S1** with initial gating on overall morphology, singlets, live cells, and CD45 positivity before proceeding with further analyses.

**Table 2:** List of antibodies used for immunophenotyping.

Antigen (mouse)	Label	Clone	Vendor	Catalog #
CD45	Brilliant Violet 605	30-F11	BioLegend	103140
CD11c	APC-eFluor 780	N418	Invitrogen	47-0114-82
CD206	Brilliant Violet 785	C068C2	BioLegend	141729
F4/80	PE/Cy7	BM8	BioLegend	123114
MHC Class II	eFluor 450	M5/114.15.2	Invitrogen	48-5321-82
Ly-6C	PE	HK1.4	Invitrogen	12-5932-82
CD11b	Alexa Fluor 488	M1/70	BioLegend	101217
Ly-6G	Alexa Fluor 647	1A8	BioLegend	127610
7-AAD	–	–	BioLegend	420403

### Production of rfhSP-D

The homotrimeric recombinant fragment of human SP-D (rfhSP-D), comprising 8 N-terminal Gly-X-Y triplets of the collagen region, α-helical coiled-coil neck region, and a C-terminal CRD, was expressed in *Escherichia coli*, purified and, characterized, as described previously (29). Endotoxin level in the rfhSP-D preparation was determined using the QCL-1000 Limulus amoebocyte lysate system (BioWhittaker Inc., USA). The amount of endotoxin in rfhSP-D preparations was found to be <4 pg/µg of rfhSP-D.

### Cell Culture

TRAMP-C2 cells were purchased from the American Type Culture Collection (ATCC) and grown in high glucose Dulbecco’s Modified Eagle Medium (DMEM) media (Gibco, USA) supplemented with 10 nm trans-dehydroandrosterone (D4000, Sigma, China), 0.005 mg/ml bovine insulin (Sigma, USA), 5% FBS (Gibo, USA), and 5% Serose (Himedia, India) at 37°C in a humidified chamber containing 5% CO_2_.

### MTT Assay

5 × 10^3^ TRAMP-C2 (passage no. 7-9) cells were plated and grown overnight in 96-well culture plates. Post-starving the cells in serum-free media for 18 h, they were treated with rfhSP-D (20µg/ml) for 24, 48, and 72 h. Cells with only culture medium served as the media control and cells treated with PBS served as the vehicle control. After incubation, 10µl MTT [3-(4, 5-dimethylthiazol-2-yl)-2, 5-diphenyltetrazolium bromide], from 5 mg/ml stock solution, was added to each well and incubated at 37^◦^C overnight. Next day, after removing the MTT solution, acidified isopropanol was added to dissolve the formazan crystals and absorbance was read at 570 nm in BioTek Synergy H1 (Winooski, VT, USA).

### Immunogenic Cell Death (ICD) Assay

5 × 10^3^ TRAMP-C2 cells were grown on coverslips overnight. Cells were incubated with rfhSP-D (20 µg/ml) or PBS (vehicle control) in a serum-free DMEM medium. After 24h treatment, the medium was removed, and cells were washed, fixed in paraformaldehyde (4% in PBS) at RT for 15 min. For intracellular high mobility group protein B1 (HMGB1) immunofluorescence, cells were permeabilized using 0.25% Triton X-100 (Sigma, USA), blocked with 5% BSA in PBST for 1h at 37° followed by overnight incubation with rabbit antibody against HMGB1 (3935S, Cell signalling Technology, 1:100 dilution) at 4°C in a humified chamber. Next day, the cells were washed and probed with Alexa Fluor 488- tagged goat anti-rabbit IgG (A11034, Invitrogen, USA, 1:400 dilution) for 1h at RT followed by staining with DAPI, and were mounted on the slides. For negative controls, only secondary antibodies were used. The fluorescence signals were observed and captured under the confocal microscope (Carl Zeiss, Germany).

Cell-surface expression of calreticulin (CRT) was evaluated with the surface-staining protocol of flow cytometry using rabbit monoclonal antibody against mouse CRT (12238S, Cell signalling Technology, 1:400 dilution in PBS) for 40 min followed by Alexa Fluor 488-tagged goat anti-rabbit IgG (A11034, Invitrogen, USA, 1:400 dilution in PBS) for 1h at RT. Similarly, intracellular CRT was evaluated by flow cytometry with staining of permeabilized cells.

To evaluate the extracellular release of HMGB1 and CRT from *ex-vivo* cultured TRAMP tumours, the culture supernatant was collected, and approx 40μl of culture supernatant was resolved on the SDS-PAGE under reducing conditions. Western blotting was performed using the above-mentioned HMGB1 and CRT antibodies (1:1000 dilution in PBS). Coomassie blue staining was used for assessing the protein load. Quantitative densitometric analysis was carried out as described above using total protein for normalization.

### Elastase-Mediated Degradation Assay

This experiment was carried out as per the previously reported protocol with some modifications (24). Briefly, 2μg rfhSP-D was incubated with 2.5μg porcine pancreas elastase (E7885, Sigma, USA) or 2.5 μg heat-inactivated elastase (HI elastase) for 30 minutes at 37 ° water bath. For heat inactivation, elastase was heated at 95° for 30 minutes before it was added to rfhSP-D. For inhibition of elastase activity, 5mM phenylmethylsulfonylfluoride (PMSF, Roche, Germany) was used. In addition, 2μg rfhSP-D was incubated with 2.5μg elastase in the presence or absence of 10mM CaCl_2_ and LPS (10μg/ml). Reactions were stopped by heating the protein sample with Laemmli buffer at 95° for 10 min. The samples were resolved on 15% SDS-PAGE under reducing conditions and the gels were Coomassie blue stained or silver stained.

### Short-Term *Ex Vivo* Culture of TRAMP Biopsies

Late TRAMP biopsies were cultured as previously described (30). Briefly, fresh tumour tissues were washed in Hanks’ Balanced Salt Solution (HBSS) (Gibco, USA), and then cut into ~1mm pieces. Tumour sections were transferred to a 24-well plate and soaked in DMEM high glucose medium (Gibco, USA) 2% with PenStrep (Sigma, USA). TRAMP tumour biopsies were divided into 4 groups: vehicle control (PBS), rfhSP-D treated (40 μg/ml), sivelestat treated (S7198, Sigma, China) (4μmol), and rfhSP-D and sivelestat treated. Biopsies were cultured for 72 hour in a culture medium at 37°C with 5% CO_2_. After treatment, biopsies were dissociated in a single cell suspension for flow cytometry. The culture supernatants were collected to evaluate the expression of HMGB1 and calreticulin *via* western blot.

### Statistical Analysis

GraphPad Prism Software version 9.00 was used for Statistical analyses. The number of technical replicates, biological replicates, and independent experiments performed are listed in the figure legends. Unpaired two-tailed Student’s t-test, one-way Analysis of Variance (ANOVA) with *post-hoc* Tukey, and two-way ANOVA with *post-hoc* Dunnett’s test were utilized as appropriate. Data are presented as mean ± standard error of the mean (S.E.M.). For all analyses, results were considered statistically significant with *P < 0.05, **P < 0.01, ***P < 0.001, and ****P < 0.0001.

## Results

### Differential Expression of Surfactant Protein-D (SP-D) in PIN and PD

**Figure 1:**
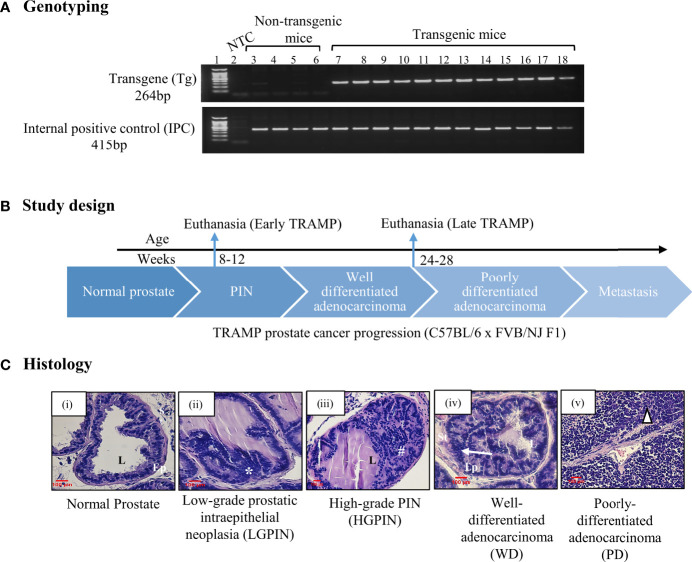
**(A)** Genotyping of TRAMP mice. PCR amplified products of murine tail DNA using rat probasin gene (Transgene; Tg)-specific primers and internal positive control (IPC) primers were run on the agarose gel to confirm the presence Tg in transgenic mice (Lane No. 7-18) whereas non-transgenic mice show no Tg-specific amplification at 264 base pairs (bp) (Lane No. 3-6), Lane 2 represents non template control (NTC). **(B)** A brief study design for sample collection. **(C)** Histology. Histological photomicrographs of normal dorsal prostate and TRAMP dorsal prostate depicting different lesions at different stages of the tumour development. (i) Normal (Control) prostate lobe with columnar epithelial lining surrounding the lumen and presence of dense stroma, (ii) Low-grade intraepithelial neoplasia (LGPIN): represents stratification of epithelial cells (*), (iii) High-grade intraepithelial neoplasia (HGPIN): represents epithelial stratification in papillary and cribriform pattern in lumen (#), (iv) Well-differentiated adenocarcinoma (WD) represents migration of epithelial cells towards smooth muscle lining of stroma (arrow), (v) Poorly differentiated adenocarcinoma (PD) represents lack of glandular architecture (triangle). L, lumen; Ep, epithelium; St, stroma. Sections stained with hematoxylin–eosin. scale =100 µm.

Using the TRAMP mice, which is an autochthonous, spontaneous, heterogeneous, and progressive, clinically relevant animal model for prostate cancer, we determined the expression of different collectin molecules throughout the tumour progression. TRAMP mice closely recapitulate the key features of human prostate cancer with respect to progression, androgen independence, and biochemical characteristics. Based on the previous reports, about 80% of C57BL/6 TRAMP mice develop prostatic intraepithelial neoplasia (PIN) by 8 weeks and more than 70% of the dorsal and lateral lobe of the prostate showed the predominant pathology of PIN at this age. The tumour progresses into poorly-differentiated adenocarcinoma (PD) by 24-28 weeks (31). Based on these characteristics of disease progression in TRAMP x FVB F1, the preclinical study was designed (**Figure 1**). TRAMP x FVB F1 mice were genotyped and grouped into non-transgenic control (lacking rat probasin gene) and transgenic TRAMP (containing rat probasin gene) (**Figure 1A**). Transgenic mice were further grouped based on their ages which reflected different stages of prostate cancer. In the first group, TRAMP males were euthanized at about 8-12 weeks (or, 2-3 months) of age to represent PIN stage, referred as ‘early TRAMP’ and tissues were collected for further analysis. The second group of transgenic males were allowed to mature and develop advanced tumours *i.e.* to the poorly differentiated adenocarcinoma (PD) stage, and euthanized at about 24–28 weeks (or, 6-7 months) of age, referred as ‘late TRAMP’ (**Figure 1B**). Age-matched non-transgenic littermates were used as controls, referred as ‘2M control’ and ‘6M control’. Haematoxylin and Eosin (H&E) staining of the dorsal prostate lobe were carried out for confirming the histological features (**Figure 1C**).

**Figure 2:**
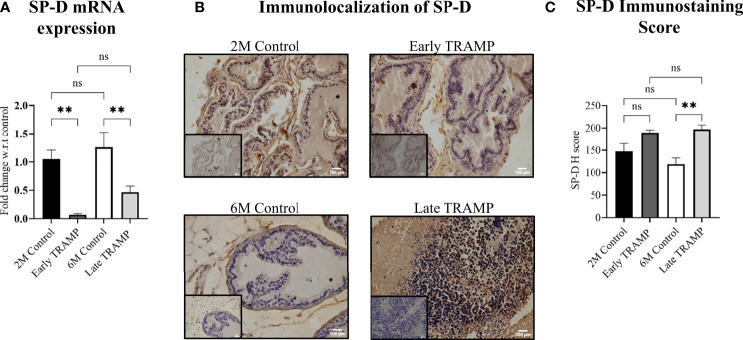
Altered expression of SP-D mRNA and protein in the early and late stages. **(A)** qRTPCR of SP-D transcript in 2M Control (n= 4), Early TRAMP (n=5), 6M Control (n=5) and Late TRAMP (n=8) normalized with GAPDH. **(B)** Representative images of SP-D immunolocalization. **(C)** Semi-quantitative immunohistochemical (IHC) analysis of SP-D protein in TRAMP (early and late) and age-matched control mice. The intensity of SP-D staining was quantitated by an open source plugin, IHC profiler compatible with open resource digital image analysis software, Image J followed by calculation of H score. For each tissue section, a minimum of three to four areas were scanned and average H score was calculated. The bar graphs represent the average H score of 2M Control (n= 3), Early TRAMP (n=3), 6M Control (n=4), Late TRAMP (n= 5) tissue samples. Inner boxes represent negative staining. Statistical significance was tested using one-way ANOVA followed Tukey’s multiple comparisons test (**p < 0.01). The data presented are mean ± SEM. ns, Non-significant.

In order to evaluate the expression of collectins in prostate tumour pathogenesis, we determined the mRNA levels of Surfactant protein-D (SP-D), Surfactant protein-A (SP-A) and Mannan-binding lectin-A (MBL-A) in the prostate of TRAMP mice during early and late stage of tumour, compared to the age-matched non-transgenic littermates (2M control and 6M control). The mRNA expression revealed significantly reduced expression of SP-D transcript ~14.87-fold in the early TRAMP and ~2.7-fold in late TRAMP when compared with control mice (**Figure 2A**). Since extrahepatic MBL-A expression has been reported in the murine testis (32) and human prostate (33), prostatic production of MBL-A transcript in TRAMP was compared with controls. MBL mRNA was not detectable in the prostate of control and TRAMP mice (**Figure S2**). Since the presence of SP-A mRNA in the human prostate has also been reported, the expression of SP-A transcript was evaluated in the murine prostate. Similar to MBL-A, SP-A expression was undetectable probably due to its lower expression in control and TRAMP prostate (**Figure S2**). None of the tested control or TRAMP mice prostate tissues had cycle threshold values <40 for SP-A mRNA.

Next, the localization of SP-D protein in prostate of early and late TRAMP was evaluated *via* immunohistochemistry. SP-D immunostaining was uniformly intracytoplasmic in TRAMP and control tissue sections. Positive immunoreactivity for SP-D was observed in the basal cell layer surrounding the acini and in the stroma of dorsal prostate of the control mice (**Figure 2B**). The expression pattern was completely altered in the poorly differentiated late TRAMP. Compared to the controls, a relatively strong homogenous staining for SP-D was detected in both early and late TRAMP mice. Densitometric analysis revealed ~1.66-fold higher SP-D immunostaining in the late TRAMP mice (**Figure 2C**).

### Increased Proteolytic Degradation of SP-D in TRAMP Prostate Tumour

**Figure 3:**
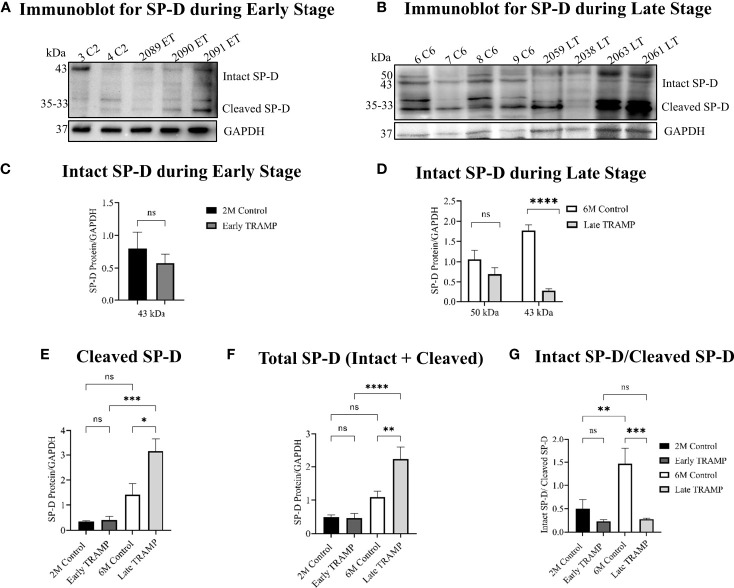
Impaired expression of the intact form of SP-D throughout the tumour progression. Total protein lysates were analysed by Western blot for SP-D and GAPDH. **(A)** Immunoblot analyses of intact monomeric form of SP-D (~45kDa) and cleaved SP-D fragments (~35kDa-~33kDa) in the Early TRAMP compared with the corresponding 2M controls. C2: 2M Control mice; ET: Early TRAMP; 3 C2: 2M Control mice no. 3; 4 C2: 2M Control mice no. 4; 2089 ET: Early TRAMP mice no. 2089; 2090 ET: Early TRAMP mice no. 2090; 2091 ET: Early TRAMP mice no. 2091. **(B)** Immunoblot analyses of intact monomeric form of SP-D (~43kDa), differentially glycosylated form of SP-D (~50kDa) and cleaved SP-D fragments (~35kDa-~33kDa) in the Late TRAMP, compared with the corresponding 6M controls. C6: 6M Control mice; LT: Late TRAMP; 6 C6: 6M Control mice no. 6; 7 C6: 6M Control mice no. 7; 8 C6: 6M Control mice no. 8; 9 C6: 6M Control mice no. 9; 2059 LT: Late TRAMP mice no. 2059; 2038 LT: Late TRAMP mice no. 2038; 2063 LT: Late TRAMP mice no. 2063; 2061 LT: Late TRAMP mice no. 2061. GAPDH was used as a loading control. **(C–G)** Densitometric analysis of proteins normalized with GAPDH. n=3-6 for each group. Data are represented as mean ± SEM. Statistical significance was determined using two-tailed Student’s t-test **(C)**; one-way ANOVA followed Tukey’s multiple comparisons test **(D–G)**, NS, Non-significant. (*p < 0.05, **p < 0.01, ***p < 0.001, ****p < 0.0001).

Pericellular proteases are reported as the key initiators of major proteolytic cascades involved in prostate tumour progression (34). Activated serine proteases are up-regulated during prostate tumour progression that can cleave SP-D to a non-functional form (23, 24, 35). Hence, it was important to evaluate if the levels of intact SP-D were altered in prostate tumour tissues due to increased proteolysis. Western blot analysis revealed less amount of the intact monomeric 43 kDa (~6.5-fold, p<0.0001) and differentially glycosylated 50 kDa forms of the SP-D protein in the late TRAMP when compared to control mice (**Figures 3B, D**). The 43 kDa form of intact SP-D protein was not significantly altered in the early TRAMP (**Figures 3A, C**). In addition, we observed a higher amount (~2.3-fold) of cleaved SP-D fragments of molecular weights ranging from ~35kDa to 33kDa in late TRAMP when compared to control mice (**Figure 3E**). Furthermore, early TRAMP prostate showed non-significant alteration in the cleaved SP-D fragments when compared to controls (**Figure 3E**). Also, there was a significant increase in the cleaved SP-D from early to advanced stage of tumour, suggesting a stage-dependent degradation of SP-D (**Figure 3E**). Similar to the IHC data, the total SP-D proteins (cleaved+intact) were significantly higher in the late TRAMP (~1.95 fold) whereas early TRAMP did not show any significant association (**Figure 3F**). Ratio of intact and cleaved SP-D was found to be significantly downregulated in late TRAMP (**Figure 3G**), suggesting increased proteolysis of SP-D protein.

### Susceptibility of rfhSP-D to Elastase-Mediated Degradation

**Figure 4:**
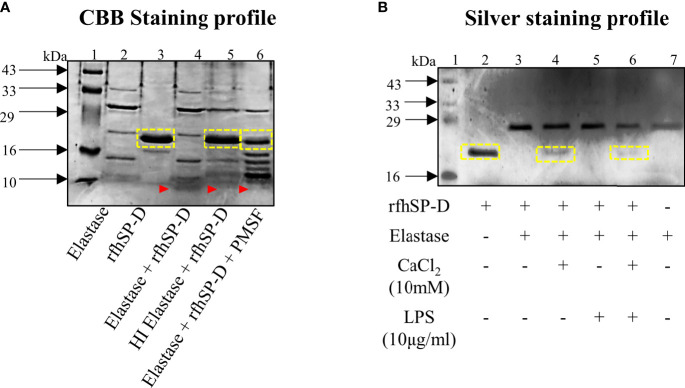
Elastase mediated degradation of rfhSP-D. **(A)** CBB-stained profile of 2μg rfhSP-D treated with 2.5μg elastase for 30 mins at 37°C. Heat-inactivated elastase was used as the negative control. Assessment of partial inhibition of elastase activity by the serine protease inhibitor PMSF (5mM). **(B)** Effect of CaCl_2_ (10mM) and LPS (10μg/ml) on elastase-mediated degradation of rfhSP-D. Yellow boxes represent band of rfhSP-D; red arrow-heads represent cleaved fragment of rfhSP-D (below 10kDa) due to elastase activity.

In view of the promising therapeutic potential of rfhSP-D in prostate cancer, it was important to know its stability in the TME. As previously reported, neutrophil elastase is one of the key serine proteases actively involved in PCa progression and is also known to cleave SP-D. Incubation of 2μg rfhSP-D with 2.5μg elastase for 30 mins at 37°C resulted in complete degradation of rfhSP-D (**Figure 4A**, Lane 4). With complete heat inactivation of the elastase at 95° for 30min, most of the rfhSP-D protein was intact with minimal degradation (**Figure 4A**, Lane 5). The proteolytic degradation products of rfhSP-D are visible below 10kDa marker (**Figure 4A**, Lane 4-6). In addition, the serine protease inhibitor PMSF (5mM), when incubated simultaneously with elastase and rfhSP-D, partially inhibited rfhSP-D degradation (**Figure 4A**, Lane 6). Further, addition of calcium chloride (CaCl_2_, 10mM) also partially inhibited elastase activity (**Figure 4B**, Lane 4). LPS did not show any effect on this degradation (**Figure 4B**, Lane 5). Thus, a specific inhibitor for elastase-mediated proteolytic activity could potentiate the impact of rfhSP-D in TME.

### Circulatory and Tumour-Derived Innate Immune Landscape Revealed Increased Elastase-Producing Immune Suppressive Cells

**Figure 5:**
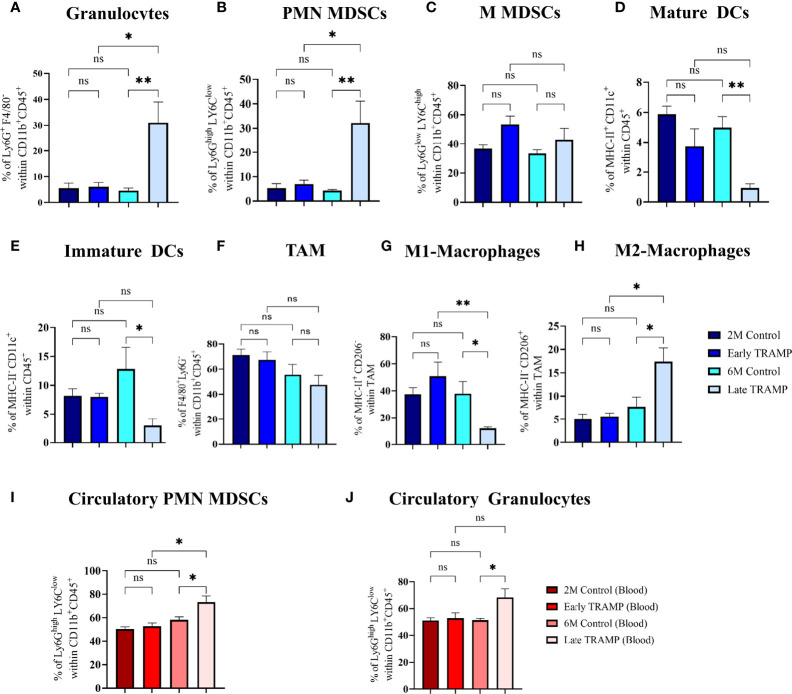
Tumour-stage-specific altered tumoural, and circulatory innate immune landscape. Control and tumour tissues were harvested at the indicated time points, single-cell suspensions were prepared and immune-phenotyped to characterize different innate immune cells like **(A)** Granulocytes (Ly6G^+^ F4/80^-^); **(B)** Polymorpho-nuclear myeloid-derived suppressor cells (PMN-MDSCs) (Ly6G^high^ LY6C^low^); **(C)** Monocytic MDSCs (MMDSCs) (Ly6G^low^ LY6C^high^); **(D)** Mature dendritic cells (DCs) (MHC-II^+^ CD11c^+^); **(E)** Immature DCs (MHC-II^-^ CD11c^+^) **(F)** Tumour associated macrophages (TAM) **(**F4/80^+^ Ly6G) **(G)** M1 macrophages (MHC-II^+^ CD206^-^ F4/80^+^ Ly6G^-^); **(H)** M2 macrophages (MHC-II^-^ CD206^+^ F4/80^+^ Ly6G^-^). Circulatory PMN-MDSCs and granulocytes were analysed using peripheral blood **(I, J).** n=4-5/group (tissue), n=5-7/group (blood)E; Data are represented as mean ± SEM. Statistical significance was determined using one-way ANOVA followed by Tukey’s multiple comparisons test, ns, Non-significant; (*p < 0.05, **p < 0.01).

Since myeloid innate immune cells are known to play an important role in solid tumours, it was important to assess their systemic and tumoural distribution in the early and advanced stages of PCa in the TRAMP model. First, we assessed tumour-infiltrating innate immune cells in the early and late TRAMP and compared it with the immune profile of control mice (**Figures 5A–H**). The gating strategies were adopted from previous studies (36, 37). Flow cytometry gating on CD45 and CD11b showed a significantly increased accumulation of granulocytes (30.98 ± 7.98%) in the late TRAMP when compared to the 6-month-old control group (4.59 ± 0.922%) (**Figure 5A**). Also, late TRAMP revealed significantly increased infiltration of the PMN-MDSCs (32.18 ± 8.945%) over control (4.354 ± 0.344%) (**Figure 5B**). While comparing early and late-stage tumours, it was observed that the late TRAMP showed a ~5.13 fold increase in granulocytes and a~4.62 fold increase in PMN-MDSCs over the early TRAMP (**Figures 5A, B**). The percentage of monocytic MDSCs (M-MDSCs) showed an increasing trend in TRAMP but could not attain a significance level (**Figure 5C**). Furthermore, the late TRAMP showed a significantly decreased abundance of both immature (2.9 ± 1.19% vs. 12.81 ± 3.76%) and mature dendritic cells (DCs) (0.98 ± 0.28% vs. 4.98 ± 0.68%) within the TME as compared to controls (**Figures 5D, E**). Tumour-associated macrophages (TAM) showed decreased accumulation of classical macrophages (M1) (12.066 ± 1.12 vs. 38 ± 8.98) and increased infiltration of alternative macrophages (M2) (17.36 ± 2.97 vs. 7.71 ± 2.06) in the late tumour as compared to control (**Figures 5F–H** **)**. A stage-dependent alteration in the M1 and M2 population between early and late TRAMP was noticed (**Figures 5G, H**). Thus, the increased percentage of granulocytes and PMN-MDSCs may serve as the main contributor to increased elastase activity which may eventually inhibit SP-D-mediated immune surveillance by degrading SP-D into a non-functional form. Increased percentage of M2 and PMN-MDSCs may also contribute to the immune-unresponsive or immunologically “cold” TME to allow immune evasion.

Next, the number of circulating myeloid cells in peripheral blood was evaluated by flow cytometry (**Figures 5I, J**). Immunosuppressive PMN-MDSCs and granulocyte populations were found to be significantly expanded in the circulation of late TRAMP as compared to the control group. The percentage of circulatory M-MDSCs did not alter significantly in TRAMP mice (**Figure S3B**). No alteration in the ratio of inflammatory and anti-inflammatory monocytes was observed in TRAMP as compared to controls (**Figure S3A**).

### Combined Treatment With rfhSP-D and Elastase Inhibitor, Sivelestat, Modulates Immune Phenotype in *Ex Vivo* TRAMP Prostate Tumour Biopsies

**Figure 6:**
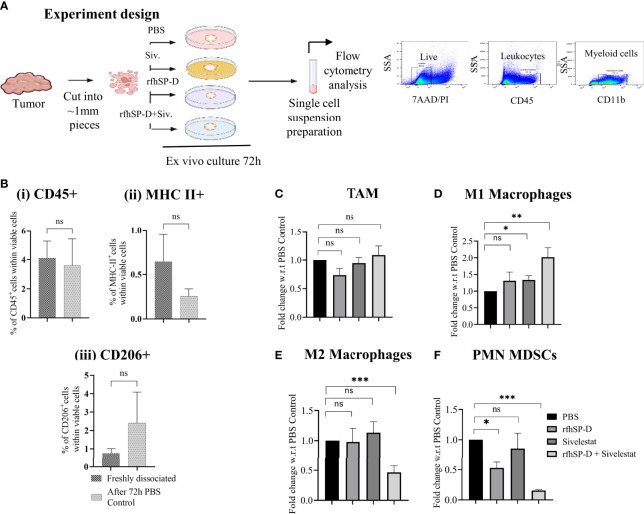
Treatment with rfhSP-D+sivelestat induced polarisation of macrophages in *ex vivo* cultured TRAMP tumours. **(A)** Study design. **(B)** Comparison of (i) CD45+, (ii) MHC-II+ and (iii) CD206+ cells percentage between freshly dissociated TRAMP tumours and tumours after 72h culture in DMEM media.**(C–F)** Tumour tissues were collected from the late TRAMP and cultured in the serum-free DMEM with PBS/rfhSP-D/sivelestat/rfhSP-D+sivelestat treatment for 72h. After treatment, single cell suspensions were prepared and percentages of different immune cell types were analysed by flow cytometry. Fold change of **(C)** tumour-associated macrophages (TAM), **(D)** M1; **(E)** M2 and, **(F)** PMN-MDSCs in *ex vivo* cultured TRAMP tumours were calculated with respect to PBS control. n=3/treatment group. Data represented as mean ± SEM. Statistical significance was determined by the two-tailed Student’s t-test. (*p < 0.05, **p < 0.01, ***p < 0.001); ns, Non-significant.

We first assessed whether *ex vivo* treatment of TRAMP biopsies with rfhSP-D and sivelestat could influence the phenotype of macrophages found in PCa. Freshly harvested TRAMP tumour biopsies were divided into 4 groups: vehicle control (PBS); rfhSP-D treated (40 μg/ml); sivelestat treated (4μmol); and rfhSP-D+sivelestat treated. Biopsies were cultured for 72 h in a standard culture medium (**Figure 6A**). Moreover, to avoid the possibility of complement activation-induced alteration of immune cell phenotype, we used serum-free medium. At the end of the treatment, biopsies were fixed and stained for immune cells markers.

As previously observed, a comparison of immune cell marker expression (CD45, MHC-II, CD206) within viable cells by flow cytometry between immediately processed tumour tissues and those cultured for 72h did not show any significant difference (**Figure 6B**) (30). We next analysed the expression of the M1 and M2 markers within the macrophage population defined as CD45^+^/CD11b^+^/F4/80^+^ Ly6G^-^ cells. The TAM population represented about 60% of the CD45^+^ leukocytes found in the biopsies (data not shown). While comparing between different treatment groups and PBS control biopsies, we observed a non-significant change in the percentage of TAM (**Figure 6C**) and a significant difference in the proportion of cells expressing the M1 and M2 markers within the TAM population. Flow cytometric analyses of tumour-derived immune cells showed that, when treated with rfhSP-D and sivelestat, the TAM skewed towards the M1 phenotype (**Figure 6D**). The treatment induced a reduction in the proportion of cells concomitantly expressing high levels of the M2-associated marker (CD206^+^ MHC-II^-^) when compared to the control PBS group (**Figure 6E**). Treatment with rfhSP-D alone or sivelestat alone did not induce such phenotypic changes in macrophages with respect to the PBS-treated control. In addition, we also observed a significant reduction in the immunosuppressive PMN-MDSCs count (CD11b^+^Ly6G^+^Ly6C^Low^) in the rfhSP-D+sivelestat treated group over the PBS control group (**Figure 6F**). Since macrophages and MDSCs are one of the major immune cells within the prostate TME, we sought to evaluate how they are modulated by rfhSP-D using short-term *ex vivo* cultured TRAMP prostate biopsies. Immunologically unresponsive “cold” TME appeared to be altered by rfhSP-D in the presence of sivelestat by polarizing TAM towards M1 phenotype; reduction in PMN-MDSCs percentage is suggestive of the immunomodulatory potential of rfhSP-D in PCa.

### Induction of Immunogenic Cell Death (ICD) by rfhSP-D

**Figure 7:**
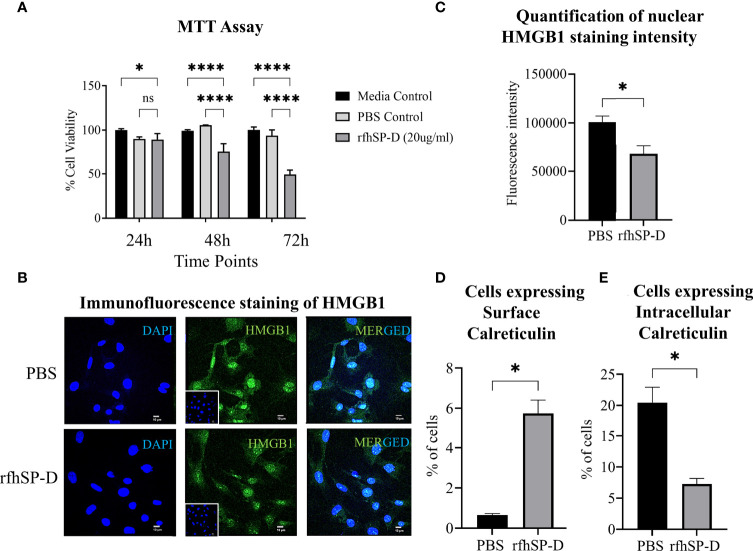
rfhSP-D induces immunogenic cell death (ICD) in TRAMP-C2 cells. **(A)** TRAMP-C2 cells were incubated in the serum-free media for 12h and then treated with or without rfhSP-D (20µg/ml in PBS). PBS was used as the vehicle control. Treatment was given for the indicated time intervals (24, 48, and 72 h). After treatment, MTT assay was done. Each bar represents % viability ± SEM of three independent experiments relative to the untreated controls or PBS control. After 24h treatment TRAMP C2 cells were analysed by immunofluorescence or flow cytometry. **(B)** Representative images showing immunofluorescence staining of nuclear high mobility group box protein 1 (HMGB1) (Green) and DAPI (Blue) **(C)** Quantification of nuclear HMGB1 staining intensity using ImageJ. **(D)** Flow cytometric analysis of cell-surface exposed calreticulin (CRT) in the non-permealized cells; and **(E)** intra-cellular calrticulin in permealized cells. Samples were analyzed in triplicate and represented as mean ± SEM. Statistical significance was determined by two-way ANOVA **(A)**, two-tailed Student’s t-test **(B–E)**. ns, Non-significant. *p < 0.05, ****p < 0.0001.

Previously, we have reported the anti-tumourigenic potential of rfhSP-D on human prostate cancer cells *in vitro* (16, 17). Interestingly, *in vitro* culture of the murine TRAMP-C2 cells with rfhSP-D (20μg/ml) resulted in cell death in a time-dependent manner and confirmed that rfhSP-D had a similar anti-tumour effect on the murine PCa cell line (**Figure 7A**). To investigate if the immunogenic cell death (ICD) contributed to this effect, HMGB1 and CRT, two major ICD damage-associated molecular patterns (DAMPs), were evaluated in TRAMP-C2 cells treated with 20μg/ml rfhSP-D for 24 hour. The rfhSP-D treated TRAMP-C2 cells showed a decreased nuclear HMGB1 staining by ~1.5 fold over PBS treated cells, signifying increased release of HMGB1 from the nucleus (**Figures 7B, C**). Percentage of cells expressing surface and intracellular CRT was evaluated by flow cytometry. rfhSP-D treatment induced a significantly increased CRT translocation to the cell surface (~8.77 fold) in non-permeabilized TRAMP-C2 and caused a ~2.8 fold reduction in the intracellular CRT expression in permeabilized cells when compared to the PBS control group (**Figures 7D, E**).

**Figure 8:**
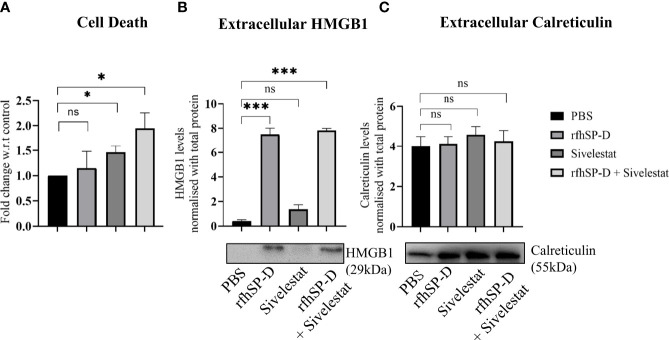
Assessment of cell death and released ICD markers in *ex vivo* cultured TRAMP tumours. Tumour tissues were collected from the late TRAMP and cultured in serum-free DMEM with PBS, rfhSP-D, sivelestat and rfhSP-D+sivelestat treatment for 72h. **(A)** After treatment, single cell suspensions were prepared and percentage of Propidium Iodide-positive (PI^+^) (dead) cells were analysed by flow cytometry. **(B, C)** After treatment, the culture-media were subjected to western blot. **(B)** Representative immunoblot and densitometric analysis of extracellular HMGB1. **(C)** Representative immunoblot and densitometric analysis of extracellular calreticulin. n=3/treatment group. Data represented as mean ± SEM. Statistical significance was determined by one-way ANOVA **(A)**, two-tailed Student’s t-test **(B, C)**. ns, Non-significant. *p < 0.05, ***p < 0.001.

Similar to the *in-vitro* observations, induction of ICD by rfhSP-D was evaluated using *ex-vivo* cultured TRAMP tumour biopsies. Flow cytometric analysis of PI+ dead cells showed a significant increase in the cell death in rfhSP-D+sivelestat group (**Figure 8A**). Extracellular expression of HMGB1 and CRT was evaluated in the culture medium of tumour explant. Both rfhSP-D treated and rfhSP-D+sivelestat treated groups showed a ~17.88 fold (p=0.003) and ~18.64 fold (p=0.002) upregulation in HMGB1 release, respectively, when compared to the PBS control (**Figure 8B**). There was no change in the extracellular CRT expression (**Figure 8C**) following treatment.

## Discussion

The role of SP-D has been extended to anti-tumoural innate immune surveillance beyond its well-established protective functions against pathogens and allergens (38). A correlation between presence/levels of SP-D transcript/protein and the cancer development was proposed recently to highlight the prognostic value of SP-D in several cancers (38). Downregulated SP-D expression has been reported in the lung, breast, and gastric cancers as compared to the healthy controls, whereas high transcriptional and protein expression of SP-D is found in the ovarian adenocarcinoma (19, 38). However, the bioinformatics studies revealed that only in the lung adenocarcinoma, SP-D mRNA had a favourable prognostic effect, whereas the presence of SP-D mRNA was negatively associated with the overall survival rate in the patients with breast, ovarian, and gastric carcinoma (38). Further, down-regulated expression of SP-D (and SP-A) at the malignant sites of human prostate adenocarcinomas as compared to the non-malignant sites and an association of weak SP-D (and SP-A) protein expression with an increasing Gleason score have been reported earlier (15). Thus, a complex regulatory mechanism involving SP-D seems to be operational in various cancers.

In order to delineate the molecular mechanisms for the initiation and progression of metastatic PCa, the TRAMP model was used as a pre-clinical autochthonous model of PCa. This genetically engineered model develops progressive, multifocal, and heterogeneous tumour which resembles various human PCa stages, thus facilitating pre-clinical studies (25, 31).

In this study, the expression of SP-D was evaluated at different stages of prostate cancer using TRAMP. Significantly downregulated transcript of SP-D was observed in both early stage (PIN: precancerous) and advanced stage (poorly differentiated adenocarcinoma; PD) of tumour development compared to age-matched non-transgenic littermates (control), a finding similar to our earlier report of a significant downregulation of SP-D mRNA in the androgen-sensitive human LNCaP cells (16). In this context, it is important to note that the glucocorticoids stimulate SP-D mRNA expression in the lung, and glucocorticoid receptor expression is low in the human prostatic carcinoma patients (39). Therefore, SP-D mRNA in the TRAMP mice could be indirectly regulated through the glucocorticoid receptor pathway in the prostate. Similarly, estrogen receptor-α was reported to stimulate SP-A expression in the fetal lung cells. In addition, a significant decrease in estrogen and androgen receptors from PIN to PD in TRAMP has been reported previously (40). Thus, it is likely that the alteration in the levels of these receptors may regulate the synthesis of SP-D in TRAMP mice. Interestingly, there was a relatively higher SP-D transcript expression in the late stage than in the early stage, most plausibly a rescue mechanism by the host. As advanced prostate tumours are histopathologically very heterogeneous (41), the expression profile of SP-D gene from late TRAMP tissues could be substantially influenced by contamination with surrounding noncancerous cells which could explain the increased expression of SP-D transcript in late TRAMP as compared to the small localized lesion of PIN or early TRAMP.

Immunolocalization in the control prostate revealed expression of SP-D in basal epithelial lining of acini and in the stromal compartment. This expression pattern was altered in the poorly differentiated late TRAMP. Thus, the considerable immunostaining of SP-D in TRAMP tumour, compared to control, may be due to its extra-prostatic origin. Since SP-D is a hydrophilic soluble PRR and is present in serum, it may circulate to the tumoural compartment as a consequence of host response (42, 43).

Tumour-infiltrating neutrophils and PMN-MDSCs are associated with a poor prognosis in many solid tumours including PCa (44). It has been observed that the stromal area of both primary and invasive PCa tissue is enriched with PMN-MDSCs more than the epithelial counterpart (45). The pro-tumourigenic effect exerted by these granulocytic myeloid cells is mainly by releasing a serine protease, neutrophil elastase (NE); importantly, these cells show a progressive increase from normal to early to advanced prostate tumours of TRAMP (34). The role of NE in promoting tumour growth, angiogenesis, and metastasis has been addressed in many cancers (46). Endocytosis of NE by the lung adenocarcinoma cells caused upregulation of PI3K-AKT survival signalling, whereas in PCa, extracellular NE triggered ERK signaling *via* the combined activation of receptor tyrosine kinases AXL and EGF (46). These pro-tumoural activities were further confirmed when inhibition of NE by the genetic deletion approach or pharmacological inhibitors showed a reduction in tumour growth in mice (46).

There are several serine proteases known to cleave SP-D. Elastase is known to cleave SP-D within the CRD region to produce a stable but non-functional 35-kDa fragment (23, 24). In the cystic fibrosis lung (35), SP-D-mediated host defense is compromised due to the increased proteolysis of SP-D. In this study, a significant increment in the cleaved SP-D and total SP-D (cleaved+intact) was found in the late TRAMP as compared to both the early TRAMP and control mice, suggesting that though the amount of total SP-D protein increased, most of it was functionally inactive. It has been shown previously that elastase-mediated degradation impairs CRD-dependent activities of SP-D can could hamper several defense mechanisms such as SP-D mediated chemotaxis of immune cells, opsonization, internalization, and killing of microbes by leukocytes (23, 24). As previously reported, immunophenotyping of the early and late TRAMP also revealed a significantly increased presence of PMN-MDSCs and granulocytes in the tumour as well as in the peripheral blood. Therefore, it is interesting to postulate that the intra-tumoural factors may be associated with an increased accumulation of MDSCs that results in the increased intra-tumoural NE level (46). Interestingly, endogenous NE inhibitors, like SERPINB1, are down-regulated in early and advanced prostate cancer, which is indicative of another strategy to establish high NE activity in tumours (41, 47). Thus, degradation of SP-D may have resulted from the plausibly increased PMN-MDSCs-derived NE activity. Several *in vitro* studies exemplified the anti-tumourigenic potential of rfhSP-D, comprising of CRD and eight Gly-X-Y repeats, but this study for the first time demonstrated that rfhSP-D is highly susceptible to the elastase-mediated degradation in tumor tissues. Hence, to maintain the stability of exogenous rfhSP-D in TME, it is important to inhibit the elastase activity.

A complex and dynamic communication between the cancer cells and immune cells within the prostate TME greatly influences tumour survival and growth. Macrophages, one of the key immune cells present within the tumour and its periphery, contribute to tumour resistance and metastases (30). The advanced TRAMP TME is marked by an abundance of immunosuppressive M2 macrophages, PMN-MDSCs with decreased M1, and mature DCs.

Hence, it is important to evaluate the anti-prostate tumour potential of SP-D in the TME. After several unsuccessful attempts to generate TRAMP-C2 syngeneic model [following reported protocols using 1x10^6^ or 5x10^6^ TRAMP-C2 cells by intraprostatic or subcutaneous routes in young (6-8 wk), and old mice (24 wk) (48, 49)] for evaluating the anti-prostate tumour effect of rfhSP-D+sivelestat *in-vivo*, we pursued the *ex-vivo* approach to evaluate the anti-tumourigenic potential of rfhSP-D. To overcome the limitations encountered with spontaneous tumour development, the usage of highly tumourigenic androgen-insensitive cell lines derived from androgen-deprived parental TRAMP-C1 or TRAMP-C2 can be used as an alternative means (50, 51).

Here we report, for the first time, the ability of rfhSP-D combined with sivelestat to modulate the immunosuppressive TME. rfhSP-D+sivelestat treated *ex vivo* cultured tumour explants showed dynamic polarization of macrophages from M2 to M1 with significant alteration in the PMN-MDSCs. Consistent with this observation, the potential of de-oligomerized SP-D in macrophages polarization was also reported in the animal model of acute kidney injury (AKI), in which induction of M1 macrophages through CRT/p38 MAPK signaling cascade was observed by the recombinant SP-D (11). The efficacy of sivelestat, in suppressing prostate as well as colon cancer xenografts in athymic mice was recently reported (44, 52). Sivelestat functions by directly inhibiting NETosis, NE-induced cancer cell proliferation, and invasion (44, 52). Here, sivelestat alone treatment resulted in significant cell death but was neither able to modulate immune profile nor HMGB1 release from the TRAMP explant. However, the impact of rfhSP-D was found to be greatly stabilized when combined with sivelestat, which suggests a new therapeutic approach for PCa (**Figure 8**). The molecular mechanisms underlying macrophage polarisation remain to be deciphered.

According to the current concept, ICD relies on the ability of specific apoptotic cancer cells treated with chemotherapeutics or radiotherapy to convey spatiotemporally coordinated release of immunogenic signals in the form of damage-associated molecular patterns (DAMPs), which provoke an active immune response (53). DAMPs exert robust immunostimulatory effects upon binding to the pattern recognition receptors (PRRs) expressed on the immune cells. The hallmarks of ICD are CRT cell surface exposure, and secretion of ATP and HMGB1 (53, 54). Here we provide evidence that rfhSP-D treatment increased the release of HMGB1 from the nucleus and a significant translocation of CRT to the cell surface, suggesting a novel mechanism through which rfhSP-D enhances immunogenicity of the tumour via ICD induction. In *ex-vivo* cultured TRAMP tumour, treatment with rfhSP-D, with or without sivelestat, induced a robust HMGB1 release in the culture medium, suggesting an enhanced ICD. Since, secreted HMGB1 is known to serve as a ligand for the receptor for advanced glycocylation end products (RAGE) and TLRs and helps in the recruitment, maturation of DCs, induction of HMGB1 by rfhSP-D may modulate immune unresponsiveness in the TME (55).

Accumulation of hyaluronic acid (HA) has been extensively reported in epithelial cancers, such as prostate, where the presence of tumour cell HA matrices is both histopathologically correlated with aggressive cancer and functionally implicated in metastatic spread (56, 57). It has been reported that HA binds to rfhSP-D and hampers its anti-apoptotic potential against the breast cancer cells (58). HA has also been reported to be involved in PCa progression, and thus, the impact of interplay between HA with SP-D in PCa is definitely worth examining. It will be interesting to analyse the impact of HA on various anti-tumour mechanisms of SP-D like EGF-EGFR interaction, ICD, and immunomodulation of rfhSP-D besides the previously reported inhibition of SP-D mediated apoptosis of cancer cells.

In summary, the present study demonstrates that the tumour milieu adversely impacts functional SP-D levels *via* reduced transcription and enhanced proteolytic degradation. We report, for the first time, an immunomodulatory role of the recombinant fragment of human SP-D (rfhSP-D) in the TRAMP tumour explants. Dynamic phenotype changes of macrophages with the downregulation of immunosuppressive PMN-MDSCs within tumour indicates a novel anti-tumour effector mechanism of rfhSP-D leading to the transformation of an immunologically unresponsive “cold tumour” into a responsive “hot tumour” microenvironment. In addition, ICD mediated by rfhSP-D is being reported in the TRAMP tumour explants for the first time. The current study provides a snapshot of the involvement of SP-D in the immunomodulation in TME of prostate cancer. Future studies would be directed to elucidate the synergistic potential of rfhSP-D and sivelestat in prostate cancer *in vivo*.

## Data Availability Statement

The original contributions presented in the study are included in the article/**Supplementary Material**. Further inquiries can be directed to the corresponding author.

## Ethics Statement

The animal study was reviewed and approved by Institutional Animal Ethics Committee (IAEC), ICMR-National Institute for Research in Reproductive and Child Health, (IAEC Project No. 09/18).

## Author Contributions

KG coordinated the study, designed, performed the experiments, analyzed the experimental data, and wrote the paper. UK provided purified and characterized rfhSP-D for the study and edited the paper. SM helped in animal handling and dissection. TM supervised the entire study, analyzed the data, and edited the paper. All authors reviewed the results and approved the final version of the manuscript.

## Funding

This work was financially supported by the Institutional Grant provided by ICMR-NIRRCH (Accession no. 1239). KG was supported by Junior and Senior Research Fellowships of the Council of Scientific and Industrial Research (CSIR, India).

## Conflict of Interest

The authors declare that the research was conducted in the absence of any commercial or financial relationships that could be construed as a potential conflict of interest.

## Publisher’s Note

All claims expressed in this article are solely those of the authors and do not necessarily represent those of their affiliated organizations, or those of the publisher, the editors and the reviewers. Any product that may be evaluated in this article, or claim that may be made by its manufacturer, is not guaranteed or endorsed by the publisher.
