# Supplementary material for: Immunomodulatory Role of Surfactant Protein-D in a Transgenic Adenocarcinoma of Mouse Prostate (TRAMP) Model
Source: Front Immunol. 2022 Jul 7;13:930449. doi: 10.3389/fimmu.2022.930449 (PMC9302643; doi:10.3389/fimmu.2022.930449)
Supplement: Supplementary file 1 [file DataSheet_1.doc]

**Immunomodulatory role of Surfactant Protein-D in a Transgenic Adenocarcinoma of Mouse Prostate (TRAMP) model**

Kasturi Ganguly^1^, Uday Kishore^2,3^, Siddhanath M Metkari^4^, Taruna Madan^1*^

**Supplementary Figures**

**
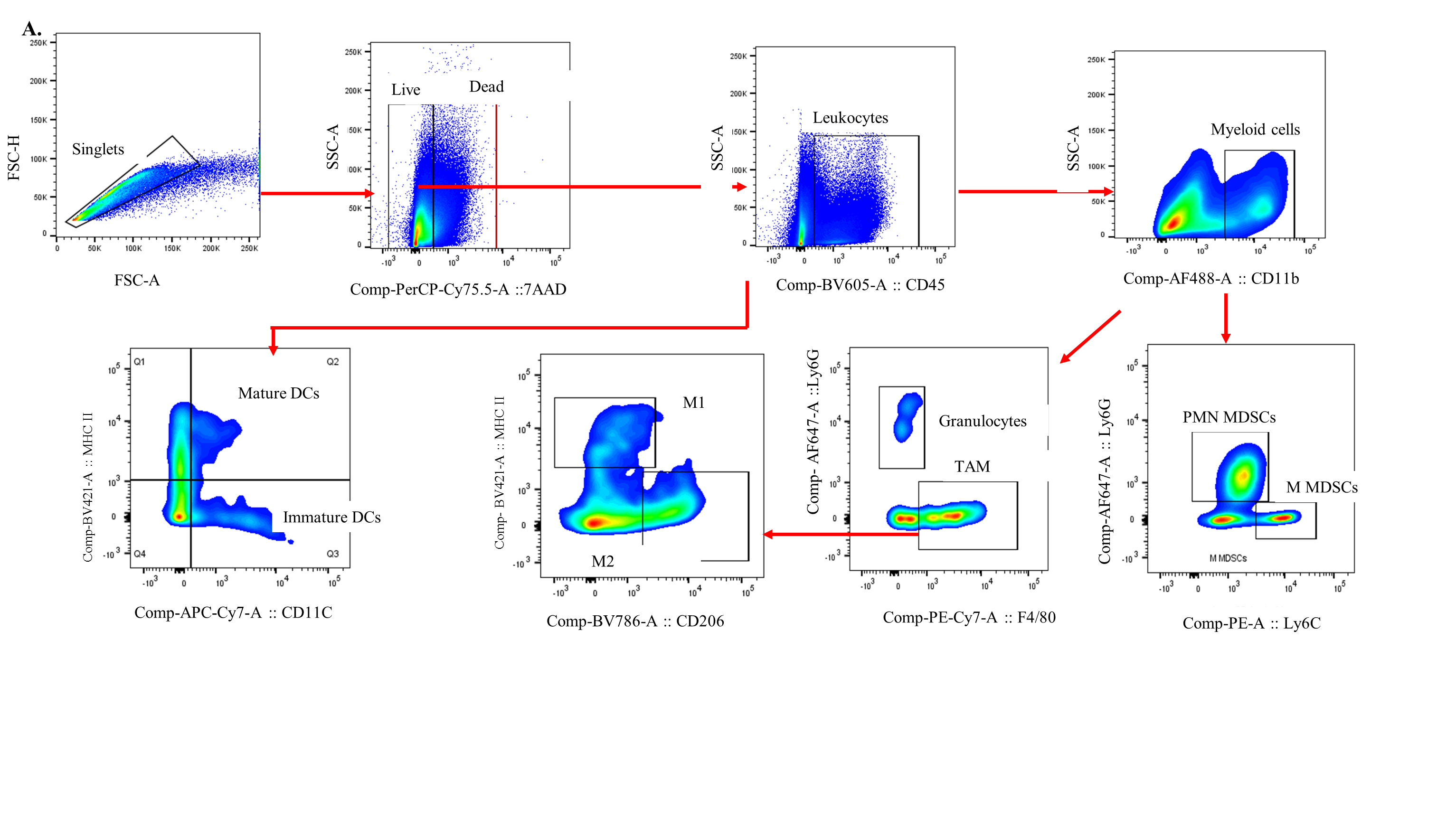

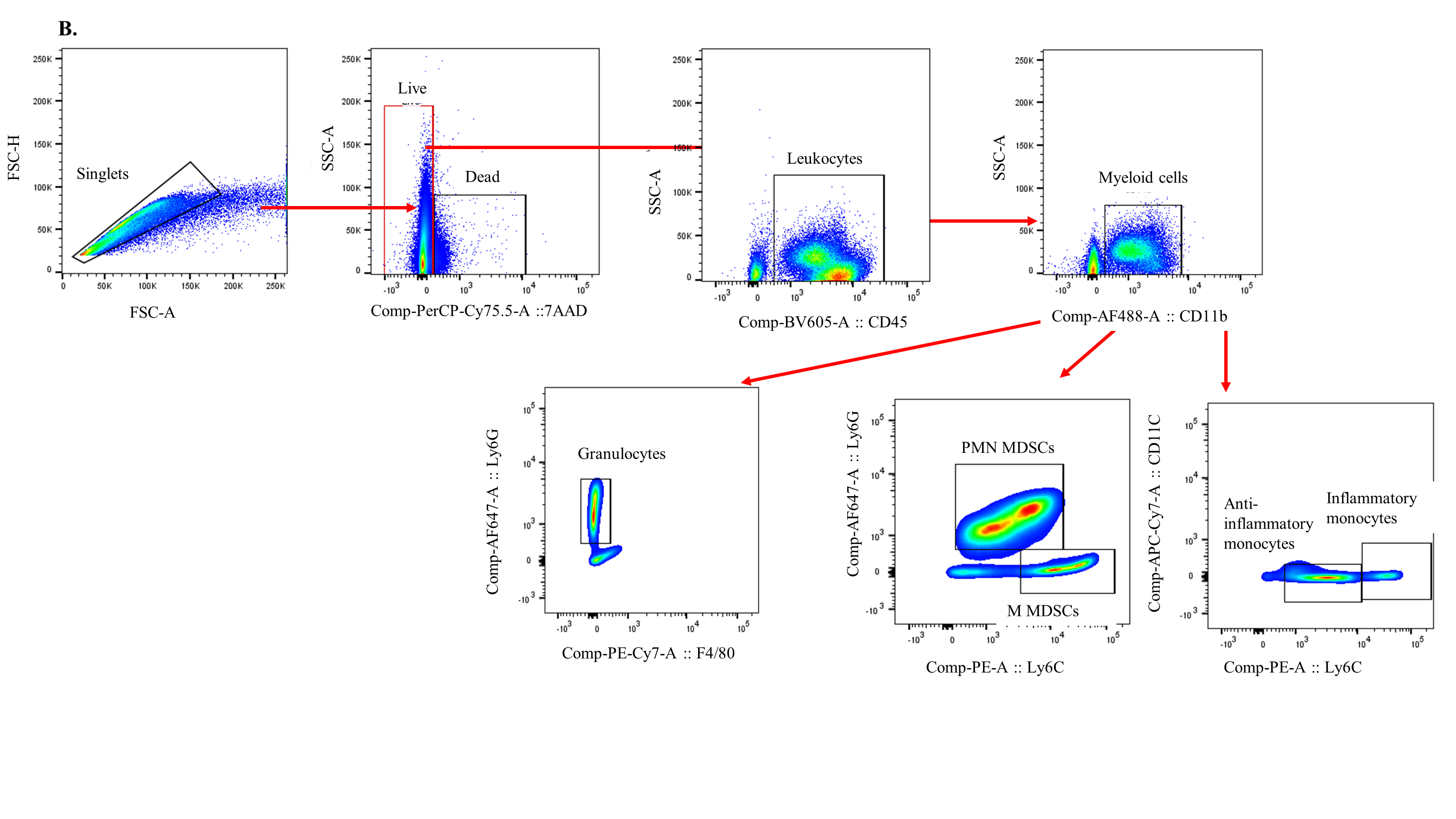
**

**Figure S1:** Flow cytometric gating strategy for **(A)** tumour-residing and **(B)** circulatory immune cells. For all the flow cytometric analyses, initial gating was performed on singlets, live cells, and CD45+ cells, followed by antigens of interest. FSC-A = Forward Scatter-Area, FSC-H = Forward Scatter-Height, SSC-A = Side Scatter-Area.


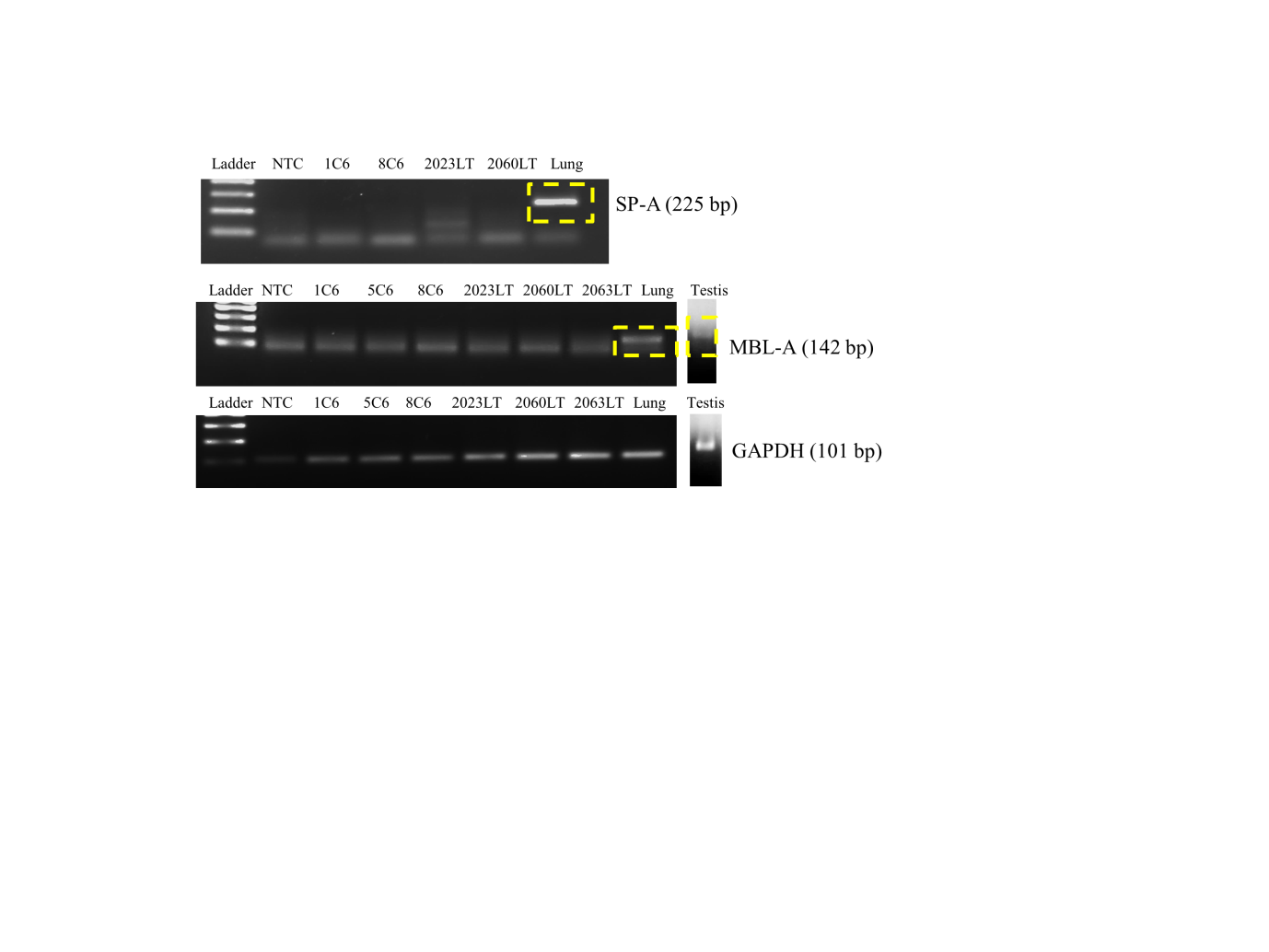


**Figure S2:** Visualization of RT-qPCR amplified transcripts of SP-A and MBL on 2% agarose gel. The positive controls *i.e* cDNA of murine lung tissue showed PCR amplified SP-A transcript at 225 base pairs and cDNA of murine testis and lung showed PCR amplified MBL-A transcript at 142 base pairs. Whereas, the control and TRAMP prostate tissue cDNAs didn’t show any specific PCR amplified SP-A or MBL-A transcript products under the same amplification conditions. GAPDH was used as an endogenous control gene for the RT-qPCR. C6: 6M Control mice; LT: Late TRAMP; 1C6: 6M Control mice no. 1; 5C6: 6M Control mice no. 5; 8C6: 6M Control mice no. 8; 2023LT: Late TRAMP mice no. 2023; 2060 LT: Late TRAMP mice no. 2060; 2063 LT: Late TRAMP mice no. 2063.


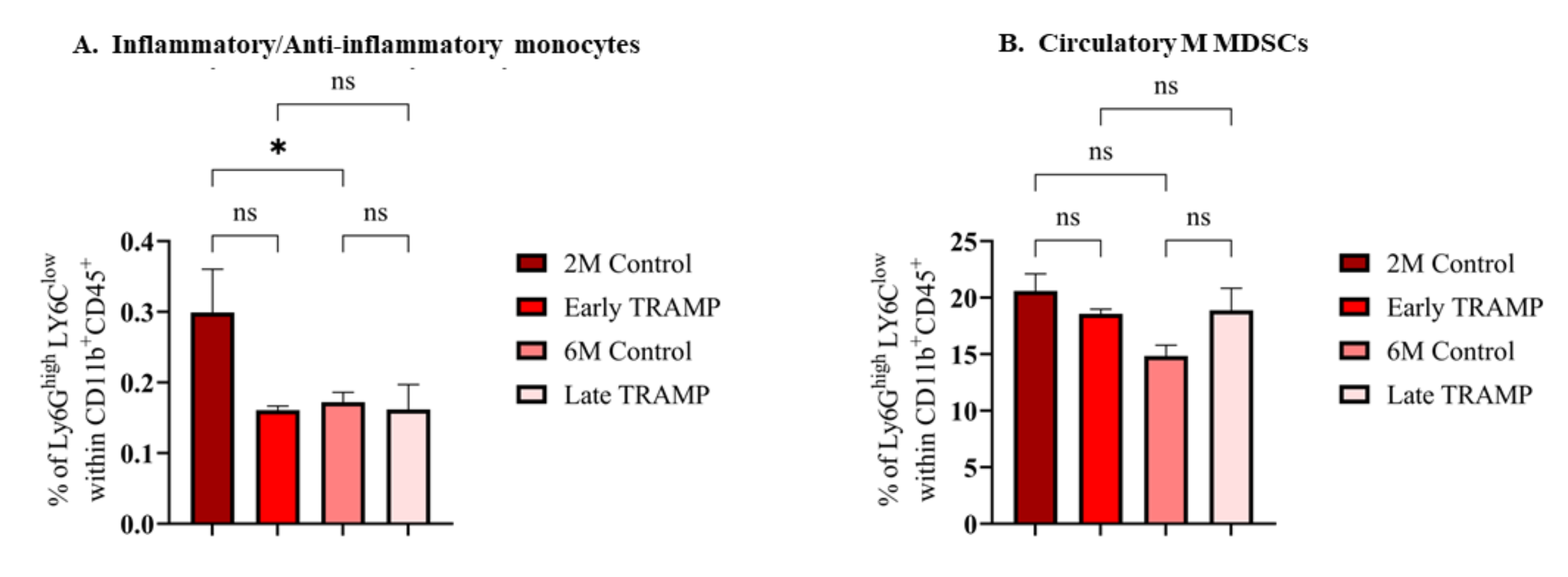


**Figure S3:** Flow cytometric analysis of the **(A)** ratio of inflammatory/anti-inflammatory monocytes and **(B)** Monocytic MDSCs (M-MDSCs) in circulation of the control and TRAMP mice. Data are represented as mean ± SEM. Statistical significance was determined using one-way ANOVA followed by Tukey's multiple comparisons test, ns: Non-significant.


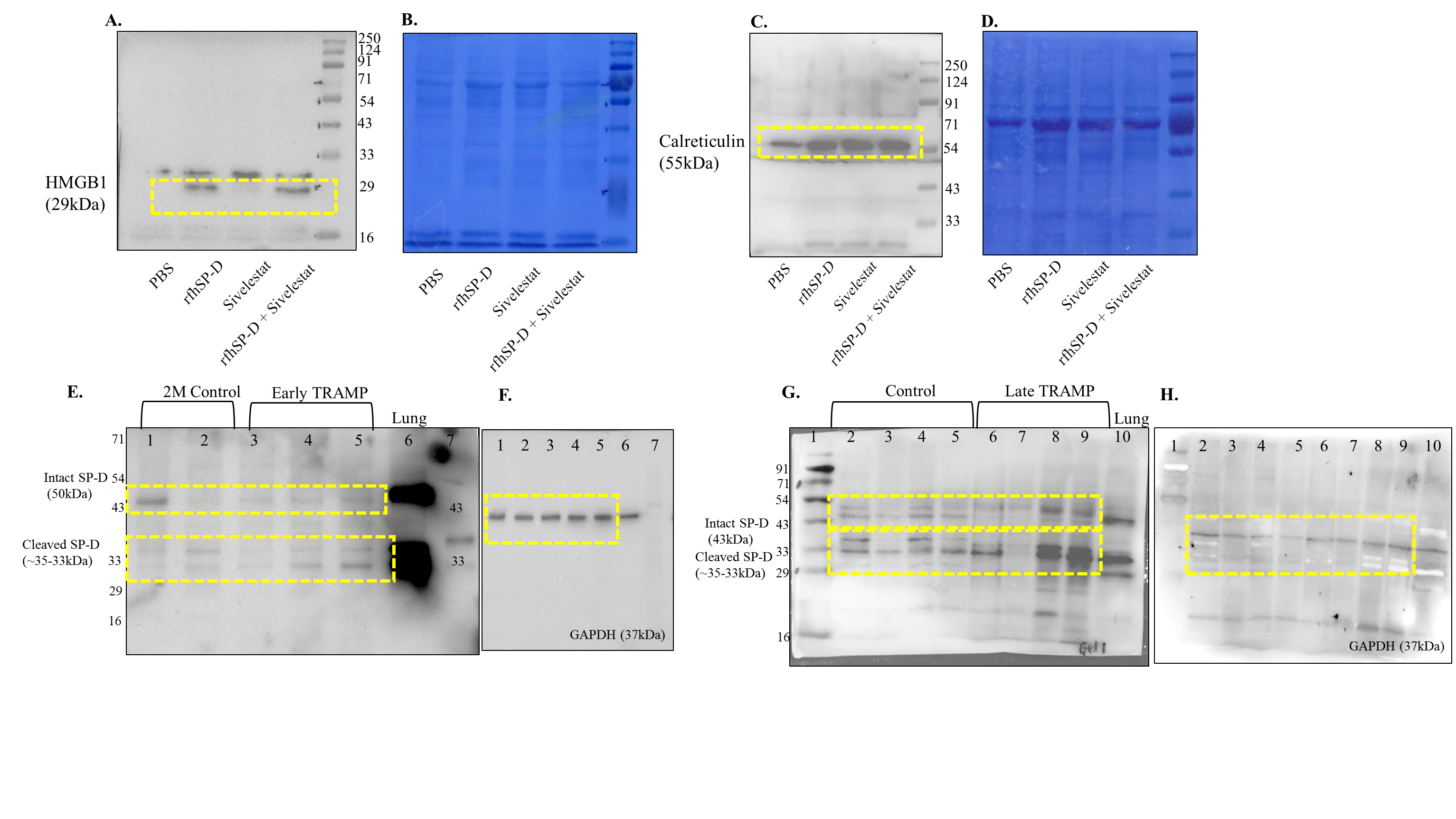


**Figure S4:** Representative uncropped images of western blots. Uncropped images of western blots from Figure 8B and 8C representing HMGB1 and Calreticulin with their respective CBB staining. **(A-D).** Uncropped images of the western blots from Figure 3A and 3B representing SP-D staining in the Early TRAMP **(E)** and Late TRAMP **(G)** with their respective GAPDH staining **(F, H)**. Lung tissue were used as a positive control for SP-D **(Lane 6 of E; Lane 10 of G).** Yellow boxes represent the cropped images displayed in main manuscript.
